# Towards the Full Realization of 2DE Power

**DOI:** 10.3390/proteomes4040033

**Published:** 2016-11-15

**Authors:** Stanislav Naryzhny

**Affiliations:** 1 Institute of Biomedical Chemistry, Pogodinskaya 10, Moscow 119121, Russia; snaryzhny@mail.ru; Tel.: +7-911-176-4453; 2 B. P. Konstantinov Petersburg Nuclear Physics Institute, National Research Center “Kurchatov Institute”, Leningrad region, Gatchina 188300, Russia

**Keywords:** two-dimensional gel electrophoresis, proteoforms, mass-spectrometry, bioinformatics

## Abstract

Here, approaches that allow disclosure of the information hidden inside and outside of two-dimensional gel electrophoresis (2DE) are described. Experimental identification methods, such as mass spectrometry of high resolution and sensitivity (MALDI-TOF MS and ESI LC-MS/MS) and immunodetection (Western and Far-Western) in combination with bioinformatics (collection of all information about proteoforms), move 2DE to the next level of power. The integration of these technologies will promote 2DE as a powerful methodology of proteomics technology.

## 1. Introduction

Currently, proteomics is a key tool in systems biology and biological studies. However, there was a delay before proteomics was launched as a method of unraveling the molecular and cellular mechanisms involved in different biological processes [1,2,3]. Upon the completion of the Human Genome Project, when genome sequencing became routine, the Human Proteome Project (HPP) was officially started at the ninth HUPO congress (2010) in Sydney, Australia. The HPP was initiated to create a comprehensive knowledge base of all human proteins. The term “proteome” was defined as the protein complement of the genome [4]. The main intricacy is that human cellular and blood plasma proteomes are tremendously complex and composed of diverse and heterogeneous gene products. These products, which are the smallest units of the proteome, are called protein species or proteoforms [5,6,7,8]. The diversity of proteoforms is generated by post-translational modifications (PTM), alternative splicing (AS), or nonsynonymous mutations (nsSNP) [9]. Global proteomics methods attempt to separate, identify, and quantify all proteoforms in a given sample. Actually, application of two-dimensional polyacrylamide gel electrophoresis (2DE) contributed to the appearance of the terms “proteome” and “proteomics” [4]. For a long time, 2DE has been not only the historic basis but also the workhorse of proteomics [10,11,12,13]. Nowadays, mass spectrometry (MS)-based systematic approaches are the main tools for studying proteome complexity. MS is the most comprehensive and versatile technique in proteomics. The ability of MS to identify and quantify thousands of proteins has had a great impact on systems biology and moves proteomics to the next level of performance [14]. Constant improvement of mass spectrometry instruments is expanding the arsenal of data acquisition and analysis approaches. A good example is the Orbitrap series, ESI LC-MS/MS instruments of high mass accuracy (less than 1 ppm), with a high resolution (up to 240,000), and a wide dynamic range (greater than 10^3^) [15,16]. Despite the high productivity, mass spectrometry in proteomics is still highly dependent on separation technologies that greatly simplify analyses of complex biological samples. A combination of MS with high-resolution protein separation is the most attractive approach for systematic investigations [17]. Many papers that describe different kinds of combinations of these systems were published [18,19,20,21,22,23]. Although there are non-gel and in-gel approaches, the main point that lies behind almost all these techniques is an application of protein or peptide separation in order to increase throughput of MS analysis. But the combination of 2DE with bottom-up mass spectrometry adds something else and looks very attractive from many points of view. Some of these points will be discussed in this review.

## 2. Power of 2DE Separation

It is interesting to trace the history of the development of various 2DE approaches for protein separation. The first 2DE technique was introduced in 1956 by Smithies and Poulik [24]. They used filter paper for the first dimension and a starch gel for the second. The method had low resolution and was not very informative. Further development and improvement of 2DE depended on continuous improvement of 1DE techniques and application of new support media for separation. Agarose gels had been used for a long time [25,26,27], but application of polyacrylamide gels considerably improved the electrophoretic separation of proteins [28]. Therefore, two dimensional separation in polyacrylamide gels of different compositions began to be applied for various investigations of ribosomal assembly and structure [29]. Further progress was made by introduction of new electrophoretic methods. Especially high resolution has been obtained by isoelectric focusing (IEF) [30,31,32,33]. The development of disc electrophoresis by Ornstain and Devis facilitated the achievement of high resolution separation according to protein size and charge [34,35]. When sodium dodecyl sulphate (SDS) was added by Laemmli into this disc electrophoresis system, it drastically improved its performance and permitted separation according to protein size only [36]. Finally, Patrick O’Farrell combined the right methods and developed his way of 2DE in 1975 [12]. The method was optimized on the basis that separations were performed according to independent parameters. The first electrophoretic separation (the first direction) was isoelectric focusing (IEF) that separated proteins due to difference in their charges or isoelectric points (pI). The second direction separation, using SDS polyacrylamide gel electrophoresis (SDS-PAGE) [36], was chosen due to its high resolution based on protein size or weight (Mw). Both methods have very good resolution, especially IEF—where resolution up to 0.001 pH units can be achieved [11,37]. The best part of this 2DE method is that the resolution obtained during IEF is not lost when the IEF gel is followed by the second direction separation (SDS-PAGE) [12]. Soon after, the method was approved as the most powerful approach for separation of complex protein samples [1,3]. Interestingly, the protocol of this approach has remained unchanged since 1975, although some improvements have been introduced. One of the improvements was the introduction of immobilized pH gradients (IPG strips) that almost completely replaced carrier ampholyte-based pH gradient in tube gels and made the life of scientists more comfortable [38,39,40]. Another improvement was the addition of new chaotropes and detergents, which have increased the solubility of hydrophobic proteins [41,42,43,44]. This demonstrates that the method was optimized by O’Farrell in the best way, from sample preparation to separation conditions. Sample fractionation has further expanded the utility of 2DE [45,46,47]. The Zoom approach and using multiple gels increased the resolution in both directions (especially IEF) [48,49,50] and achieved better separation of proteoforms. Using multiple IPGs covering very narrow, overlapping pH ranges enabled detection of more than 5500 protein spots in a 2DE gel of *Escherichia coli* lysate by colloidal Coomassie G-250 staining [51]. An assembled 93 cm × 103 cm cyber gel allowed detection of about 11,000 spots in a 2DE gel from primary cultured rat hippocampal neurons by autoradiography [48,52]. Concerning the number of spots, we should discuss two important issues in 2DE—resolution and sensitivity. If we consider 2DE just as a separation method, we should talk about the resolution only. This issue can be improved considerably by adjustment of the first and second dimensions of 2DE. Spot detection is the second step and depends considerably on the staining method used. There are diverse demands for this step, such as sensitivity, linearity, and compatibility with downstream processes such as mass spectrometry. This step is thoroughly described in many practical books or reviews devoted to 2DE [53,54,55,56,57]. Two commonly used protein stains are Coomassie brilliant blue (R250, R350, G250) and silver nitrate. Coomassie staining has a moderate sensitivity (ng level) but good linearity and accuracy. The dye binds to basic and aromatic amino acids by electrostatic and hydrophobic interactions and is compatible with subsequent MS analyses. Silver staining is more sensitive than Coomassie (pg level) but has worse linearity and accuracy [58]. It is also poorly adapted for MS analyses, as proteins can be cross-linked when formaldehyde is used as a reductant. Though adjustments were performed [59], silver staining is not very good for MS, making the Coomassie the preferred stain for proteomics. Sensitive techniques (ng–pg level) based on fluorescent stains (like Sypro Ruby or Flamingo) are also available and can be considered as a possible compromise. These staining methods combine high sensitivity, linearity, and compatibility with mass spectrometry [60]. The detection can be done in non-covalent and covalent binding mode [61]. In addition, the use of covalently bound fluorescent probes that differ mainly by their excitation and emission wavelengths allows to perform multiplexing of samples on 2DE gels. This technique, called difference gel electrophoresis (DIGE), allows the user to perform 2DE of several samples in the same gel [62]. Protein identification is an extra step and is performed by immunostaining or mass spectrometry. Accordingly, in 2DE-based proteomics, there are several steps: sample preparation, 2DE separation, detection, quantification, and identification [63]. In this chain of procedures, the bottleneck is the spot detection (staining) sensitivity but not 2DE itself [64,65]. However, many people still consider 2DE as a comprehensive process that combines separation and detection of protein spots as well.

## 3. Decoding of Information Hidden inside the 2DE Gel

Separation with high resolution is the main function of 2DE and just the first step towards obtaining necessary information about all proteoforms. The next important step is the identification of proteoforms. Interestingly enough, this step was the main reason why separation of total tissue proteins was frequently criticized by protein biochemists. They argued (even Patrick O’Farrell) that separation of total proteins was useless since it was impossible to identify and characterize all of them [11]. At that time (the late 1970s and early 1980s), it was true, since this step was one of the bottlenecks of the procedure, and only the identification of proteins of special interest was possible [63]. Edman sequencing or immunostaining was used for protein identification. An excellent example is the study of proliferation cell nuclear antigen (PCNA). It took several years and considerable efforts of several labs to characterize just one spot on the 2DE map [66,67,68,69]. Immunological methods were mainly used for this purpose. They are still successfully used in 2DE-based proteomics. The detailed and deep 2DE analysis actually revealed the presence of many PCNA proteoforms [69,70,71,72].

A more sophisticated immunological approach in combination with 2DE (Far-Western) can give an extra dimension to already multi-dimensional proteome analysis [73]. It is used to study protein–protein interactions. In this case, the proteins after 2DE separation are transferred to a membrane and treated with a set of buffers, which apply washes with SDS solutions and allows prey proteins to denature and renature [73,74,75]. The membrane is then blocked and probed with a purified bait protein. The bait protein is detected on spots where the prey protein is located if the bait proteins and the prey protein bind together. Antibodies can be used to detect the bait proteins [73,74].

**Figure 1:**
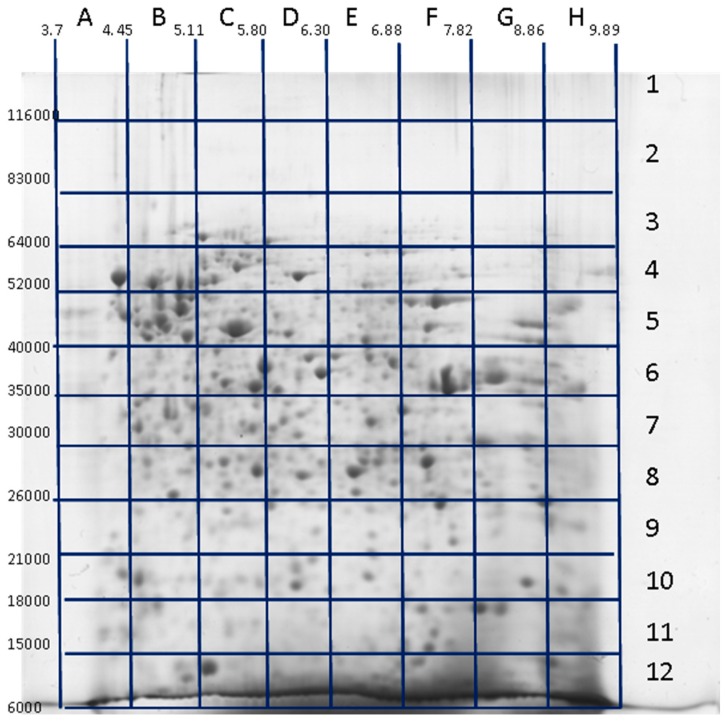
Identification of proteoforms located in different sections of 2DE (two-dimensional electrophoresis) gel (“pixel-picking” approach). Glioblastoma cell extract was applied for the run. After separation, the gel was stained with Coomassie R250, and the 2DE map was calibrated according to the positions of several previously detected major protein spots. The gel was divided into 96 sections, identified as 1–12 along the Mw dimension (vertical) and A–H along the pI dimension (horizontal). All these gel sections were cut, treated with trypsin according to protocol for mass spectrometry, and the peptides were analyzed by ESI LC-MS/MS. Adapted from [90].

**Figure 2:**
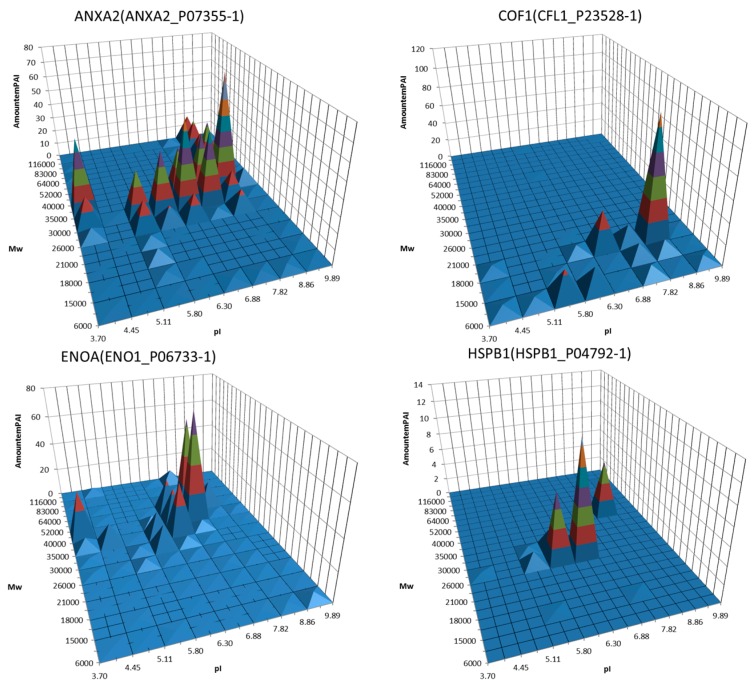

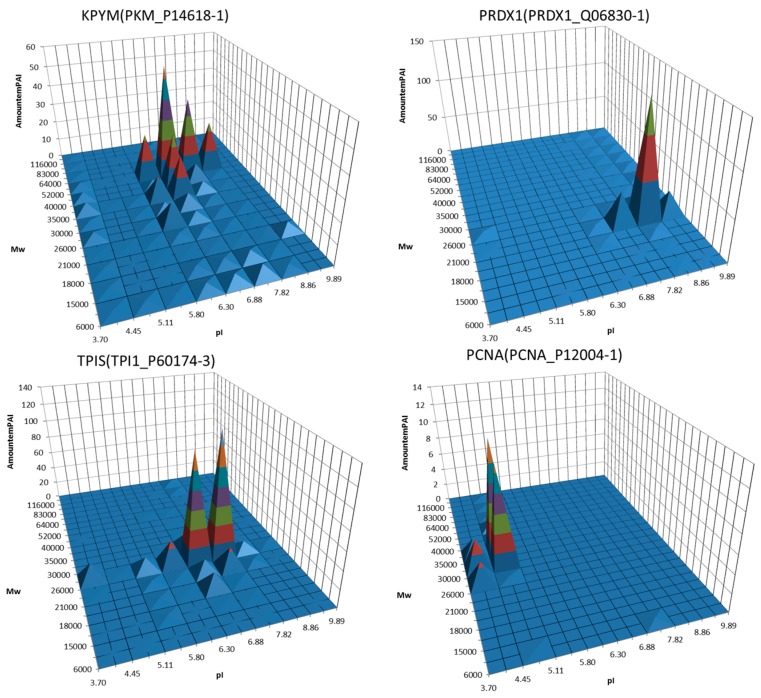
Examples of 3D graphs showing distribution of proteoforms between different sections of the 2DE map. A semi quantitative (estimated by emPAI) distribution of the same protein (gene) around the different gel sections was plotted. Proteins, the potential biomarkers, are shown. Adapted from [90].

All the techniques mentioned above are very informative but also tedious and not convenient for a large-scale analysis. The situation was radically changed when MS became a central element for proteomics analysis [76,77]. The major steps of the 2DE-based proteomics became: (1) sample preparation/extraction; (2) protein separation by 2DE; (3) protein spot detection and quantitation; (4) computer-assisted analysis of 2DE patterns; (5) protein identification and characterization; and (6) 2DE protein database construction. The mass spectrometry approach to identify proteins by searching for the best match between peptide masses produced by specific hydrolysis and peptide masses calculated from theoretical cleavage of proteins in the appropriate sequence database was developed simultaneously by different groups [78,79,80,81,82]. The peptide mass sets were acquired by Matrix-Assisted Laser Desorption/Ionization Time-of-Flight Mass Spectrometry (MALDI-TOF MS). This approach was named peptide mass fingerprinting (PMF), and has been the simplest and most powerful technique for high-throughput protein identification for a long time. The overall approach has been used to generate annotated 2DE gel databases for many cell types (www.lecb.ncifcrf.gov/EP/table2Ddatabases.html). The main idea behind this approach, namely, with only one protein in each spot as the basis, worked well only with pure proteins. It also seems that this scheme worked well in the case of uncomplicated proteomes, such as mycoplasma, for instance, where the expected numbers of proteoforms in proteomes were not so great as in mammalian cells [83,84,85,86]. After application of more sophisticated MS instruments for identification of proteins in 2DE spots (especially ESI LC-MS/MS), it was revealed that, depending on the gel resolution, the spots often contained more than a single protein, especially in the case of mammalian cells [87,88]. So, the quantitation of proteins became an ambiguous task. It was found that the majority of spots contained a single, most abundant proteoform, but many of them were composed of various proteoforms. In this case, the densitometry of spots cannot be used for accurate proteoform quantitation and special MS techniques need to be applied. In addition, not all proteoforms of a given sample are revealed, thus leading to a loss of information. Only the proteins detected as spots after staining are cut out and analyzed. This dilemma can be solved only by analyzing all parts of the gel [89,90]. Using this approach, we are not losing any proteoform that was separated by 2DE and present inside the gel. The general representation of this technique is shown in Figure 1. The main steps were as follows: first, 2DE separation was performed. The gel was stained with Coomassie R350, scanned, and the image produced (2DE map) was analyzed by Image Master 2DE Platinum (GE Healthcare, Pittsburgh, PA, USA). The map was calibrated according to the position of several major protein spots that had been previously identified: actin cytoplasmic (ACTB_HUMAN, pI 5.29/Mw 42,052), 78 kDa glucose-regulated protein (GRP78_HUMAN, pI 5.07/Mw 72,333), tropomyosin alpha-3 chain (TPM3_HUMAN, pI 4.68/Mw 32,950), stathmin 1 (STMN_HUMAN, pI 5.76/Mw 17,292), and alpha-enolase (ENOA_HUMAN, pI 7.01/Mw 47,481). Next, the gel was divided into 96 sections and identified as 1–12 along the Mw dimension and A–H along the pI dimension (Figure 1). According to the calibration, each section was given pI/Mw coordinates. All these gel sections were cut and treated with trypsin according to the protocol for mass-spectrometry by ESI LC-MS/MS. The tryptic peptides obtained from each 2DE section were separated using reversed-phase nano-LC gradients and analyzed online by Orbitrap Q-Exactive mass spectrometer. Finally, protein identification and relative quantification were performed using Mascot “2.4.1” and emPAI. In total, up to 500 unique proteins were identified in each section by Mascot search. All proteins detected in the same section were given the pI/Mw parameters of this section. Accordingly, the same proteins detected in different sections were considered as different proteoforms. About 20,000 proteoforms coded by ~4000 genes were identified using this approach [89,90]. Moreover, information about the set of proteoforms coded by each of these genes can be extracted and represented separately as a 3D chart (Figure 2). Considering that only 96 sections from a small gel (8 cm× 8 cm) were taken for analysis, the situation can be improved significantly by increasing the gel size, the sample loading, and the number of sections. With the use of this approach, the problem of staining sensitivity can be resolved. However, there are still some issues that need to be tackled. One of them is a resolution issue, which drops largely if we cut the gel into big sections. The best choice is to cut the gel into pieces with dimensions bordering the smallest spots. However, another issue arising in this case is the productivity of ESI LC-MS/MS. Though this type of mass spectrometry has high throughput it also has time frames. Usually it takes 0.5–1 h to run one sample. Accordingly, we will need about one week of continuous work on the Orbitrap to analyze 96 samples from a single 2DE gel. Another problem is proteoform quantitation. A quantitation parameter often used, the exponentially modified form of protein abundance index (emPAI) [91] used in the papers [89,90] is not ideal, since it gives only a relative and not very accurate estimation of protein content. Hopefully, the situation here will be improved, keeping in mind the rapid progress in mass spectrometry. Selected reaction monitoring (SRM) and isotope-coded affinity tag (ICAT) are excellent examples of the power of MS technology in protein quantitation [92,93,94]. There are also other approaches that are described and reviewed [95].

## 4. Utility of 2DE Separation Parameters

In addition to its high resolution, 2DE’s separation parameters, isoelectric point, and molecular weight (pI and Mw) are the second advantage. As both separation directions (first—IEF and second—SDS-PAGE) are performed in denaturing conditions, all complexes, quaternary and tertiary structures, are disrupted, and polypeptides are unfolded. For the first direction, the denaturation is provided with a buffer containing chaotropic agents and nonionic detergents (lysis buffer: 7 M urea, 2 M thiourea, 4% CHAPS, 1% dithiothreitol (DTT), 2% ampholytes, pH 3–10, and protease inhibitor mixture) [85]. After IEF, each polypeptide resides at the pH position that corresponds to its pI value. To perform the second direction, the proteins are prepared for transfer initially by treatment with an equilibration buffer containing the anionic detergent SDS and DTT (equilibration solution: 50 mM Tris, pH 8.3, 6 M urea, 2% sodium dodecyl sulfate (SDS), 30% glycerol, and 1% dithiothreitol (DTT)). Further, incubation is followed by equilibration in the same solution but containing 5% (*w*/*v*) iodoacetamide instead of DTT. Iodoacetamide alkylates cysteines that are reduced by DTT and prevents their reoxidation to form disulfides. The gel for the second direction also contains SDS (0.1%), so the proteins are present in denaturing environments all the time. SDS not only denatures proteins, but binds to them by hydrophobic forces. The number of SDS molecules is roughly proportional to the polypeptide’s length (or its weight, as it is unfolded). Since the SDS molecules are negatively charged, all of the polypeptides will have approximately the same charge-to-weight ratio (around 1.4 g SDS/1 g protein). Additionally, SDS’s charge swamps the inherent charge of the protein and gives every protein the same negative charge-to-weight ratio, allowing proportional migration from cathode to anode. Thus, the polypeptides become rod-like structures possessing a uniform charge density that is the same net negative charge per unit weight. Moreover, the rate of this migration will depend only on the size (weight) of the protein, as the separating gel has sieving properties (the resistance to the motion of a particle increases with particle size). The electrophoretic mobility is a linear function of the logarithm of the molecular weight [96]. The use of protein standards allows calibration of the gel and calculation of each polypeptide’s weight. As a result, after a 2DE run, a protein map can be produced, where the coordinates of each spot correspond to the isoelectric point (pI) and molecular weight (Mw) of a particular polypeptide. Although simplified, this situation is correct for the vast majority of proteins. Actually, there are some exceptions, which are discussed below.

## 5. 2DE Databases

An additional advantage of the 2DE method is that it can be used not just for separation but for database creation. In this case, it could be possible to separate all proteoforms by 2DE, produce an interactive map of spots (proteoforms), give proteoforms the coordinates on a 2DE map, and deposit all this information about the proteins in a sample (proteoforms). This is the basic idea that lies behind the creation of the 2DE-based databases [97,98,99]. Thus, this approach has been widely used in many labs, permitting numerous proteins to be identified on two-dimensional polyacrylamide gel electrophoresis (2D PAGE) maps, and from this several 2D PAGE databases were built. Reference protein maps have been established for proteins of human (plasma, red blood cells, liver, cerebrospinal fluid, and other human samples and cell lines), rat, mouse, *Arabidopsis thaliana*, *Dictyostelium discoideum*, *Escherichia coli*, *Saccharomyces cerevisiae*, and *Staphylococcus aureus* origins [2,100,101,102,103,104,105,106,107,108,109,110]. In 1993, the annotated SWISS-2DPAGE database was developed to organize and gather all these data about proteins identified on various 2DE maps [97]. A molecular biology server called ExPASy was set up at Geneva University Hospital on a Sun Microsystems SPARC Station-2. Remote access to the SWISS-2DPAGE protein map database was organized through the ExPASy server. Additionally, very soon an idea of a federated 2DE database that offers an easy and efficient way to publish and share 2DE data was realized. Taking advantage of the World Wide Web, this approach allows each laboratory to maintain their own database and interconnect it with other similar databases [99].

## 6. Virtual 2DE

It is important that pI and Mw, which are the principal parameters of a polypeptide, can also be calculated based on just the sequence of the polypeptide. Using a series of well-characterized peptides, Bjellqvist et al. determined the pK value of all amino acids in similar experimental conditions [111]. They showed that the isoelectric points of the polypeptides calculated based on their amino acid sequences matched well the experimentally determined pI values. A theoretical molecular weight can be calculated by summing the molecular weights of the amino acids composing the sequence, then subtracting (nAA − 1) × 18.015, where nAA is the number of amino acids in the sequence and 18.015 is the molecular weight of water. As the theoretical molecular weights of polypeptides usually match the experimental Mw calculated, based on migration in SDS-PAGE gels [96], it provides a possibility for prediction of the position of the known proteins on the two-dimensional gel. Thus, an idea of a virtual, or pseudo, 2D gel map came to our attention. Several different programs such as Virtual Gel or JVirGel were developed [112,113], allowing the calculation of theoretical molecular weights and presentation on a 2DE map (pseudo 2DE map) for every known polypeptide sequence. Some of them just build a virtual 2DE gel from a list of proteins with the given pI/Mw or sequences [112,114,115]. Such an attractive visual representation was used as a platform for manipulation and analysis of different types of data available (bioinformatics resources) for particular proteins. For instance, ProtPlot (initially VIRTUAL2D) was developed by Medjahed et al. to provide tools for the mining of quantified virtual 2D gel (pI, Mw, expression) data of estimated expression from the CGAP EST mRNA tissue expression database [113,116,117]. Data analysis was applied to the differential expression of gene products in pooled libraries from normal and cancer tissues [117]. The pI/Mw parameters can be computed not only for a polypeptide’s canonical (nonmodified) sequences but also for post-translationally modified forms as well. The ProMoST program can calculate pI/Mw taking into account such modifications as phosphorylation (presence of phosphoserine/threonine (S/T), phosphotyrosine (Y)), deamidation of Asparagine (N) or Glutamine (Q), or blockage of terminal ends [118,119].

It seems appropriate to mention that the term “virtual 2DE” was also coined earlier for the approach, where MALDI-TOF mass spectrometry was substituted for SDS-PAGE size-based separation. In this case, software is used to transform and assemble the mass spectra and IEF into a virtual 2D gel. Use of MALDI-TOF for the second dimension provides advantages in mass resolution, mass accuracy, and sensitivity over classical 2D gels [120,121,122].

## 7. Virtual-Experimental 2DE

Another way to achieve better representation and usage of bioinformatics and experimental data is to combine virtual and experimental 2DE. Actually, this approach was partially applied to compile the SWISS-2DPAGE database when the database was introduced. The computed (theoretical) pI and Mw were used for some proteins to check their possible position on the map. The detected spots of human heat shock protein 60 or HSP60 were used as an example of correlation of theoretical and experimental data [97]. If a protein was not identified in SWISS-2DPAGE, then only the boxes were drawn, showing the region around the theoretical pI and Mw of the protein, where the given protein was possibly located. The aspects of processing and posttranslational modifications should also be considered. For example, if a protein is known to contain a signal sequence and/or a propeptide, the extent of these regions should be considered, when computing pI/Mw. If a protein is acetylated or phosphorylated, the possible location box is expanded towards the left side (acidic direction) of the main box, extending the region in which the protein is expected to be present because of additional negative charge. In the case of glycosylation, a dashed region is additionally added, extending up, because of the relatively high weight of added glycans [97,123].

Rapid development in ESI LC-MS/MS, especially Orbitrap technology, is accompanied by production of a large amount of data [16]. Here, the combination of 2DE with ESI LC-MS/MS looks like a good choice, since together they allow better representation of the information obtained [88,89]. In one example [88], proteins (cell extract and human blood plasma) were run by 2DE. After staining and protein spot identification by MALDI-TOF MS (one spot—one protein), the protein maps with pI/Mw coordinates were generated. Next, the spots were further analyzed by ESI LC-MS/MS and all the proteoforms present in each spot were identified. The theoretical pI/Mw of identified proteins was calculated using Compute pI/Mw [124] or obtained from NextProt database. The relationship between theoretical and experimental parameters was analyzed, and the correlation plots were built. Additionally, virtual/experimental information about different protein species/proteoforms from the same genes was extracted. This analysis showed that the major proteoforms detected in the cell line have pI/Mw parameters similar to the theoretical values. In contrast, the experimental pI/Mw of minor proteoforms was found to be very different from the theoretical pI/Mw parameters. A similar situation was observed in plasma to a much higher degree. It means that the minor protein species are heavily modified in the cellular proteome and even more in the plasma proteome [88]. A better view can be obtained by graphs, where experimentally measured physicochemical parameters of proteoforms are plotted against the theoretical (in silico) parameters of master forms of the corresponding proteins. A master form is a proteoform that has a canonical sequence [125]. A master protein is the primary translation product of the coding sequence. According to UniProt, the choice of canonical sequence is based on at least one of the following criteria: a) it is the most prevalent, b) it is the most similar to orthologous sequences found in other species, and c), by virtue of its length or amino acid composition, it allows the clearest description of domains, isoforms, polymorphisms, post-translational modifications, etc. In the absence of any other information, the longest sequence is considered as canonical [88]. Using this virtual/experimental approach, any difference between experimental and theoretical parameters is a basis for determining its origin. Actually, it can happen because of several reasons. Reason #1—another splice variant rather than a canonical sequence is expressed in a particular proteome (shift can be observed in pI and Mw directions). Reason #2—the canonical sequence is modified by PTMs (mostly pI shift is observed, since usually there is only a small change of Mw because of PTMs). A common modification such as phosphorylation adds 80 Da to the mass of polypeptide, while acetylation adds 42 Da. There are other popular modifications, like ADP-ribosylation or glycosylation, which can produce an Mw shift in addition to a pI shift. Glycosylation provides greater proteomic diversity than any other PTMs. This PTM is characterized by various glycosidic linkages, including N-, O- and C-linked glycosylation, glypiation (GPI anchor attachment), and phosphoglycosylation. In case of multiple modifications, we usually see a chain of spots on the 2DE map. Reason #3—proteolytic processing or degradation. In the case of processing, usually only a single N-terminal peptide is cleaved, but in the case of degradation (by exo- or endoprotease) multiple fragments are produced. Reason #4—exceptions or abnormal migration of the polypeptide in SDS-PAGE. The most notable example here is cellular tumor antigen p53. The molecular weight of the canonical isoform of p53 alpha (master protein) is 43,653 Da, but in SDS-PAGE it migrates in the area of 53 kDa. This is the reason why this protein was called p53. The cause of anomalous migration is a high number of proline residues in the protein, which slows its migration on SDS-PAGE [126]. The mechanism of this action is not completely clear. A possible answer is that prolines have a fixed angle that causes bending of the denatured polypeptide. Because of this, the protein will have a more extended “random coil” conformation rather than “rod conformation” and therefore have lower mobility in SDS-PAGE. Other technical reasons for abnormal protein migration (non-reduced S–S bonds, non-complete denaturing, etc.) are also possible. They should be taken into account before coming to a conclusion. Regardless, using this virtual/experimental representation, we can combine all available information about proteoforms into virtual/experimental 2DE protein databases.

## 8. Conclusions

Now, we can stress some main points about 2DE and its application. 2DE analyses have several advantages. First, 2DE can be used to analyze complex samples without preliminary steps. Second, it separates not just proteins but proteoforms (protein species), according to their pI/Mw parameters, and enables the large-scale analysis in top-down proteomics (study of intact proteoforms). Third, it can be used for comparing theoretical and experimental information and database organization. Fourth, it is visual and provides a final analytical quantitative image, representing the protein heterogeneity in the sample of interest. Fifth, the proteoform diversity can be further studied by various techniques compatible with 2DE, including immunodetection (Western, Far-Western) or MS.

Also, taking into account recent publications [88,89], we can reconsider the major steps in 2DE-based proteomics. In the near future, they would be

(1) sample preparation/extraction
(2) protein separation by 2DE
(3) protein gel staining (Coomassie)
(4) proteoform location, identification, characterization, and quantitation by ESI LC-MS/MS
(5) virtual/experimental 2DE protein database construction

It is evident that spot detection and spot quantitation are not included in the list. Instead, mass spectrometry can perform this work, and provide direct information for proteoforms rather than for spots. However, we need MS instrumentation of a very high efficiency for the best performance of these experiments. In this case, 2DE/MS combination will constitute a highly robust and practical approach that will meet the requirements of proteomics in panoramic analysis. This approach may potentially combine the benefits of “top-down” and “bottom-up” proteomics and provide the valuable information in basic and applied (especially health-oriented) studies.
